# Isoniazid Inhibits the Heme-Based Reactivity of *Mycobacterium tuberculosis* Truncated Hemoglobin N

**DOI:** 10.1371/journal.pone.0069762

**Published:** 2013-08-01

**Authors:** Paolo Ascenzi, Andrea Coletta, Yu Cao, Viviana Trezza, Loris Leboffe, Gabriella Fanali, Mauro Fasano, Alessandra Pesce, Chiara Ciaccio, Stefano Marini, Massimo Coletta

**Affiliations:** 1 Interdepartmental Laboratory of Electron Microscopy, University Roma Tre, Roma, Italy; 2 Institute of Protein Biochemistry, National Research Council, Napoli, Italy; 3 Department of Biology, University of Roma Tor Vergata, Roma, Italy; 4 Department of Sciences, University Roma Tre, Roma, Italy; 5 Department of Structural and Functional Biology and Center of Neuroscience, University of Insubria, Busto Arsizio (Varese), Italy; 6 Department of Physics, University of Genova, Genova, Italy; 7 Department of Clinical Sciences and Translational Medicine, University of Roma “Tor Vergata”, Roma, Italy; 8 Interuniversity Consortium for the Research on the Chemistry of Metals in Biological Systems, Bari, Italy; Institut de Pharmacologie et de Biologie Structurale, France

## Abstract

Isoniazid represents a first-line anti-tuberculosis medication in prevention and treatment. This prodrug is activated by a mycobacterial catalase-peroxidase enzyme called KatG in *Mycobacterium tuberculosis*), thereby inhibiting the synthesis of mycolic acid, required for the mycobacterial cell wall. Moreover, isoniazid activation by KatG produces some radical species (*e.g.*, nitrogen monoxide), that display anti-mycobacterial activity. Remarkably, the ability of mycobacteria to persist *in vivo* in the presence of reactive nitrogen and oxygen species implies the presence in these bacteria of (pseudo-)enzymatic detoxification systems, including truncated hemoglobins (trHbs). Here, we report that isoniazid binds reversibly to ferric and ferrous *M. tuberculosis* trHb type N (or group I; Mt-trHbN(III) and Mt-trHbN(II), respectively) with a simple bimolecular process, which perturbs the heme-based spectroscopic properties. Values of thermodynamic and kinetic parameters for isoniazid binding to Mt-trHbN(III) and Mt-trHbN(II) are *K* = (1.1±0.1)×10^−4^ M, *k*_on_ = (5.3±0.6)×10^3^ M^−1^ s^−1^ and *k*_off_ = (4.6±0.5)×10^−1^ s^−1^; and *D* = (1.2±0.2)×10^−3^ M, *d*_on_ = (1.3±0.4)×10^3^ M^−1^ s^−1^, and *d*_off_ = 1.5±0.4 s^−1^, respectively, at pH 7.0 and 20.0°C. Accordingly, isoniazid inhibits competitively azide binding to Mt-trHbN(III) and Mt-trHbN(III)-catalyzed peroxynitrite isomerization. Moreover, isoniazid inhibits Mt-trHbN(II) oxygenation and carbonylation. Although the structure of the Mt-trHbN-isoniazid complex is not available, here we show by docking simulation that isoniazid binding to the heme-Fe atom indeed may take place. These data suggest a direct role of isoniazid to impair fundamental functions of mycobacteria, *e.g.* scavenging of reactive nitrogen and oxygen species, and metabolism.

## Introduction

Tuberculosis (TB) affects about 15 million people, and there are about 9 million new cases per year. Note that about 3% of all newly diagnosed patients are affected by multidrug-resistant TB (MDR-TB) and extensively drug-resistant TB (XDR-TB). The vast majority of the world burden of tuberculosis is in developing countries (*i.e.*, in South-East Asia and sub-Saharian Africa regions), which is one of the main reasons why only 23% of the prevalent active cases are currently estimated to receive an appropriate anti-tuberculosis treatment. Although effective antimicrobial strategies have been established, about 1.7 million people still die every year by tuberculosis. Recently, the emergence of antibiotic resistant strains of *Mycobacterium tuberculosis* (*M. tuberculosis*) and the high incidence of new mycobacterial diseases among immunocompromised patients has led to new research priorities to combat mycobacteria [1]–[10].

Although host genetic factors may probably contribute, the incomplete and inadequate treatment is the most important factor leading to the development of MDR-TB and XDR-TB [7], [9]–[15]. The selection and transmission of multidrug-resistant tuberculosis indicates the resistance to at least isoniazid and rifampicin, the two fundamental components of any regimen for the treatment of drug-susceptible tuberculosis [16]–[19]. In the treatment of MDR-TB, residual first-line drugs, such as ethambutol, pyrazinamide, and streptomycin must be appropriately combined with additional second-line drugs (*e.g.*, aminoglycosides (including streptomycin), capreomycin, *p*-aminosalicylic acid, thioamides, ryphamycins, fluoroquinolones, linezolid, clarithromycin, beta-lactams, clofazimine, phenothiazines, nitroimidazopyrans, and cycloserine), guided by individual susceptibility patterns [13], [17], [18], [20]. XDR-TB is resistant to rifampicin and isoniazid, two so-called “first-line” antituberculosis drugs, in addition to any antibiotic from the fluoroquinolone group, and at least one of the three injectable anti-tuberculosis drugs (amikacin, capriomycin, and kanamycin) [15], [17], [21], [22]. The management of MDR-TB and XDR-TB is a challenge which should be undertaken by experienced clinicians at centres equipped with reliable laboratory service for mycobacterial culture and *in vitro* sensitivity testing as it requires prolonged use of expensive second-line drugs with a significant potential for toxicity [6], [10], [13], [17], [20], [23]–[25].

Isoniazid has a key role in TB prevention and treatment, being bactericidal to rapidly-dividing mycobacteria, but bacteriostatic if they are slowly-growing [5], [17], [18], [26], [27]. However, isoniazid is never used alone to treat active tuberculosis because resistance quickly develops [5], [16], [18], [27], [28]. Interestingly, although isoniazid displays an antidepressant effect(s) [29]–[32], drug-associated psychosis has been reported [33]–[37].

Isoniazid is a prodrug that is activated by a mycobacterial catalase-peroxidase enzyme that in *M. tuberculosis* is called KatG. This enzyme couples the isonicotinic acyl with NADH to form the isonicotinic acyl-NADH complex that binds tightly to the enoyl-acyl carrier protein reductase known as InhA, thereby inhibiting the recognition of the natural enoyl-AcpM substrate. This process impairs the synthesis of mycolic acid, required for the mycobacterial cell wall [26], [38], [39]. The most common mechanism of isoniazid resistance is represented by mutations in KatG that decrease its activity, preventing the conversion of the prodrug isoniazid to its active metabolite [38], [40]. Other mechanisms of resistance are related to a mutation in the mycobacterial *inhA* and *KasA* genes involved in mycolic acid biosynthesis [41]–[43] and mutations in NADH dehydrogenase [28], [44].

Isoniazid activation by KatG produces radical species that display anti-mycobacterial activity; in particular, nitrogen monoxide is generated from the oxidation of hydrazide nitrogen atoms by *M. tuberculosis* KatG [45]. Isoniazid-derived nitrogen monoxide inhibits *M. tuberculosis* growth *in vitro*, likely through the impairment of the cytochrome *c* oxidase activity. Accordingly, nitrogen monoxide scavengers, like 2-(4-carboxyphenyl)-4,4,5,5-tetramethylimidazoline-1-oxyl-3-oxide, provide protection against the anti-mycobacterial activity of isoniazid. Moreover, it has been proposed that mycothiol, which is an actinobacterial thiol composed by a Cys residue with an acetylated amino group linked to glucosamine, which is then linked to inositol, acts as a nitrogen monoxide trap to form S-nitrosomycothiol. However, S-nitrosomycothiol can be deleterious *to M. tuberculosis* as it can transnitrosylate a variety of intracellular targets [26], [46]. Thus, isoniazid-derived nitrogen monoxide is likely to act in synergy with other isoniazid-derived species to contribute to overall activity of the drug [45]–[48]. Therefore, the ability of mycobacteria to persist *in vivo* implies the presence in these bacteria of (pseudo-)enzymatic detoxification systems, including truncated hemoglobins (trHbs) [49]–[55].

Here, we report that isoniazid binds reversibly to ferric and ferrous *M. tuberculosis* trHb type N (or group I; Mt-trHbN(III) and Mt-trHbN(II), respectively) with a simple bimolecular process, which perturbs the heme-based spectroscopic properties. Accordingly, isoniazid inhibits azide binding to Mt-trHbN(III), Mt-trHbN(III)-catalyzed peroxynitrite scavenging, and Mt-trHbN(II) oxygenation and carbonylation. Docking simulation shows that isoniazid may bind to the heme-Fe atom with different geometries, which imply ligand-linked structural changes of the heme pocket. These data suggest a direct role of isoniazid to impair fundamental functions of mycobacteria, *e.g.* scavenging of reactive nitrogen and oxygen species and oxygen metabolism.

## Materials and Methods

### Materials

Recombinant wild-type ferrous oxygenated Mt-trHbN (Mt-trHbN(II)-O_2_) was expressed and purified as described elsewhere. The Mt-trHbN(II)-O_2_ concentration was determined using the value of the molar absorptivity in the Soret region, ε_416 nm_ = 1.07×10^5^ M^−1^ cm^−1^ [56].

The ferric derivative of Mt-trHbN (Mt-trHbN(III)) was prepared by oxidation of Mt-trHbN(II)-O_2_ with a 10-fold excess of potassium ferricyanide. Once the reaction was completed, ferri/ferrocyanide was removed from the Mt-trHbN(III) solution by desalting it over a HiTrap desalting column prepacked with Sephadex G-25 Superfine (purchased from Amersham Pharmacia Biotech Italia, Cologno Monzese, MI, Italy) equilibrated with 5.0×10^−2^ M phosphate buffer (pH 7.0).The Mt-trHbN(III) concentration was determined using the value of the molar absorptivity in the Soret region, *i.e.* ε_406 nm_  = 1.41×10^5^ M^−1^ cm^−1^ [57].

The ferrous deoxygenated derivative of Mt-trHbN (Mt-trHbN(II)) was prepared by reduction of either Mt-trHbN(II)-O_2_ or Mt-trHbN(III) with sodium dithionite (final concentration, 1.0×10^−2^ M). The Mt-trHbN(II) concentration was determined using the value of the molar absorptivity in the Soret region, *i.e.* ε_432 nm_  = 1.03×10^5^ M^−1^ cm^−1^ [56], [57].

CO was purchased from Linde AG (Höllriegelskreuth, Germany). The CO solution was prepared by keeping in a closed vessel the 1.0×10^−1^ M phosphate buffer solution (pH 7.0) under CO at *P* = 760.0 mm Hg and 20.0°C, anaerobically. The solubility of CO in the aqueous buffered solution is 1.03×10^−3^ M, at *P* = 760.0 mm Hg and 20.0°C [58].

All the other products were from Merck AG (Darmstadt, Germany). All chemicals were of analytical grade and were used without further purification.

### Rapid-mixing instruments

Rapid-mixing experiments were performed using either the SX18.MV stopped-flow apparatus (Applied Photophysics, Salisbury, United Kingdom) or the SFM-20 stopped-flow apparatus (Bio-Logic SAS, Claix, France); the dead time was 1.0 ms.

### Isoniazid binding to Mt-trHbN(III)

Thermodynamics and kinetics of isoniazid binding to Mt-trHbN(III) were analyzed in the framework of the minimum reaction mechanism depicted by Scheme A:
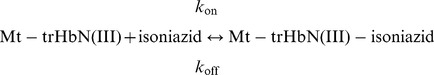
(A)


Values of the dissociation equilibrium constant (*i.e.*, *K*  =  *k*_off_/*k*_on_), of the second-order association rate constant (*i.e.*, *k*_on_), and of the first-order dissociation rate constant (*i.e.*, *k*_off_) for isoniazid binding to Mt-trHbN(III) were obtained spectrophotometrically between 350 nm and 460 nm, at pH 7.0 (1.0×10^−1^ M phosphate buffer) and 20.0°C.

The value of *K* was determined by adding small aliquots of the isoniazid stock solution (8.0×10^−3^ M) to the Mt-trHbN(III) solution (4.0×10^−6^ M). The drug-dependent absorbance changes of Mt-trHbN(III) were recorded after incubation of 10 min after each addition. Test measurements performed between 10 min and 2 h of Mt-trHbN(III)-drug incubation excluded slow kinetic effects. Isoniazid binding to Mt-trHbN(III) was analyzed by plotting values of the molar fraction of the Mt-trHbN(III)-drug complex (*i.e.*, α) *versus* the free drug concentration (*i.e.*, [isoniazid]), according to Equation 1 [58]:
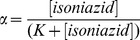
(1)


Values of the apparent pseudo-first order rate constant for isoniazid binding to Mt-trHbN(III) (*i.e.*, *k* ^obs^) were determined by rapid-mixing the isoniazid and Mt-trHbN(III) stock solutions (8.0×10^−3^ M and 4.0×10^−6^ M, respectively) and plotting the data according to Equation 2 [58]:
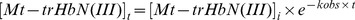
(2)


Values of *k*_on_ and *k*_off_ for isoniazid binding to Mt-trHbN(III) were determined by plotting values of *k* ^obs^ *versus* the free drug concentration (*i.e.*, [isoniazid]) according to Equation 3 [58]:
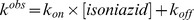
(3)


### Effect of isoniazid on azide binding to Mt-trHbN(III)

Thermodynamics and kinetics of azide binding to Mt-trHbN(III), in the absence and presence of isoniazid, were analyzed in the framework of the minimum reaction mechanism depicted by Scheme B:
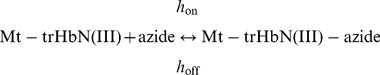
(B)


Values of the dissociation equilibrium constant (*i.e.*, *H*  =  *h*_off_ */h*_on_), of the second-order association rate constant (*i.e.*, *h*_on_), and of the first-order dissociation rate constant (*i.e.*, *h*_off_) for azide binding to Mt-trHbN(III) were obtained spectrophotometrically between 350 nm and 460 nm, at pH 7.0 (1.0×10^−1^ M phosphate buffer) and 20.0°C.

The value of *H* was determined by adding small aliquots of the azide stock solution (8.0×10^−3^ M) to the Mt-trHbN(III) solution (4.0×10^−6^ M). The azide-dependent absorbance changes of Mt-trHbN(III) were recorded after incubation of 10 min after each addition. Test measurements performed between 10 min and 2 h of Mt-trHbN(III)-azide incubation ruled out slow kinetic effects. Azide binding to Mt-trHbN(III) was analyzed by plotting values of the molar fraction of the Mt-trHbN(III)-azide complex (*i.e.*, α) *versus* the free ligand concentration (*i.e.*, [azide]) according to Equation 4 [58]:
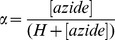
(4)
*H* changes to *H* ^obs^ in the presence of isoniazid.

Values of the apparent pseudo-first order rate constant for azide binding to Mt-trHbN(III) (*i.e.*, *h* ^obs^) were determined by rapid-mixing the azide and Mt-trHbN(III) stock solutions (8.0×10^−3^ M and 4.0×10^−6^ M, respectively) and plotting the data according to Equation 5 [58]:
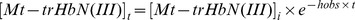
(5)


Values of *h*_on_ and *h*_off_ for azide binding to Mt-trHbN(III) were determined by plotting values of *h* ^obs^ *versus* the free ligand concentration (*i.e.*, [azide]) according to Equation 6 [61]:
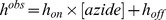
(6)


Thermodynamics of competitive inhibition of azide binding to Mt-trHbN(III) by isoniazid were analyzed in the framework of the minimum reaction mechanism depicted by Scheme C [59]:
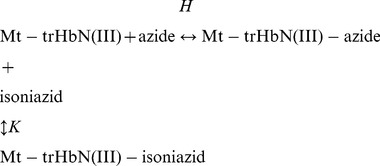
(C)


Values of the dissociation equilibrium constant for azide binding to Mt-trHbN(III) in the presence of isoniazid (*H* ^obs^) were obtained at [isoniazid]  = 1.0×10^−3^ M, 2.0×10^−3^ M, and 4.0×10^−3^ M, at pH 7.0 (1.0×10^−1^ M phosphate buffer) and 20.0°C.

The inhibitory effect of isoniazid on azide affinity for Mt-trHbN(III) was analyzed by plotting values of the *H* ^obs^/*H* ratio *versus* the free drug concentration (*i.e.*, [isoniazid]) according to Equation 7 [59]:
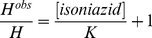
(7)


### Peroxynitrite isomerization by Mt-trHbN(III) in the absence and presence of isoniazid

Kinetics of peroxynitrite isomerization by Mt-trHbN(III) in the absence and presence of isoniazid was recorded spectrophotometrically at 302 nm (ε_302 nm_  = 1.705×10^3^ M^−1^ cm^−1^) [60]–[67] in the absence and presence of Mt-trHbN(III) (final concentration 2.5×10^−6^ – 1.0×10^−5^ M) and isoniazid (final concentration 3.0×10^−5^ – 4.0×10^−4^ M) by rapid mixing the Mt-trHbN(III) or buffer solution with the peroxynitrite solution (final concentration 2.5×10^−4^ M).

Kinetics of peroxynitrite isomerization by Mt-trHbN(III) in the absence and presence of isoniazid was analyzed in the framework of the minimum reaction mechanism depicted by Scheme D [60]–[67]:
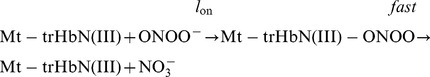
(D)


Values of the pseudo-first-order rate constant for Mt-trHbN(III)-mediated peroxynitrite isomerization (*i.e.*, *l* ^obs^) were determined in the absence and presence of isoniazid according to Equation 8 [60]–[67]:
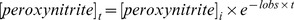
(8)


Values of the second-order rate constant for Mt-trHbN(III)-mediated peroxynitrite isomerization (*i.e.*, *l*_on_) and of the first-order rate constant for peroxynitrite isomerization in the absence of Mt-trHbN(III) (*i.e.*, *l*_0_) were determined from the linear dependence of *l* ^obs^ values on the Mt-trHbN(III) concentration according to Equation 9 [60]–[67]:
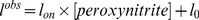
(9)


Values of *l*_0_ for peroxynitrite isomerization in the absence of Mt-trHbN(III) were also determined in the absence and presence of isoniazid from the analysis of the time-dependent absorbance decrease at 302 nm according to Equation 10 [60]–[67]:
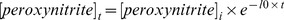
(10)


The value of *K* for isoniazid binding to Mt-trHbN(III) was determined from the dependence of *l*_on_ on the drug concentration (*i.e.*, 3.0×10^−5^ M ≤ [isoniazid] ≤4.0×10^−4^ M) according to Equation 11 [60]–[67]:
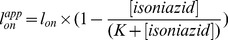
(11)where *l*_on_^app^ is the value of *l*_on_ in the presence of isoniazid.

The levels of NO_2_^–^ and NO_3_^–^ obtained by peroxynitrite isomerization in the absence and presence of Mt-trHbN(III), isoniazid, and azide were determined spectrophotometrically at 543 nm by using the Griess reagent and VCl3 to catalyze the conversion of NO_3_^–^ to NO_2_^–^, as described previously [60], [62], [68]. Calibration curves were obtained by measuring four to eight standard sodium nitrite and sodium nitrate solutions in 1.0×10^−1^ M phosphate buffer, pH 7.0 and 20.0°C. The samples were prepared by mixing 500 μL of a Mt-trHbN(III) solution (initial concentration 1.0×10^−4^ M in 2.0×10^−1^ M phosphate buffer, pH 7.0) with 500 μL of a peroxynitrite solution (initial concentration 4.0×10^−4^ M in 0.01 M NaOH) with vortexing, at 20.0°C, in the absence and presence of isoniazid (1.0×10^−2^ M) and azide (1.0×10^−2^ M). The reaction mixture was analyzed within approximately 10 min.

### Isoniazid binding to Mt-trHbN(II)

Thermodynamics and kinetics of isoniazid binding to Mt-trHbN(II) were analyzed in the framework of the minimum reaction mechanism depicted by Scheme E:
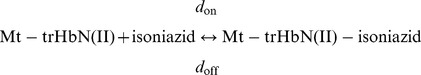
(E)


Values of the dissociation equilibrium constant (*i.e.*, *D*  = *d*_off_/*d*_on_), of the second-order association rate constant (*i.e.*, *d*_on_), and of the first-order dissociation rate constant (*i.e.*, *d*_off_) for isoniazid binding to Mt-trHbN(II) were obtained spectrophotometrically between 375 nm and 460 nm, at pH 7.0 (1.0×10^−1^ M phosphate buffer) and 20.0°C, in the presence of dithionite ( =  1.0×10^−2^ M; *i.e.*, under anaerobic conditions).

The value of *D* was determined from the dependence of the total amplitude of kinetics of isoniazid binding to Mt-trHbN(II) on the ligand concentration. Isoniazid binding to Mt-trHbN(II) was analyzed by plotting values of the molar fraction of the Mt-trHbN(II)-drug complex (*i.e.*, α) *versus* the free drug concentration (*i.e.*, [isoniazid]) according to Equation 12 [58]:
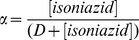
(12)


Values of the apparent pseudo-first order rate constant for isoniazid binding to Mt-trHbN(II) (*i.e.*, *d* ^obs^) were determined by rapid-mixing the isoniazid and Mt-trHbN(II) stock solutions (*i.e.*, 2.0×10^−1^ M and 3.0×10^−6^ M, respectively) and plotting the data according to Equation 13 [58]:
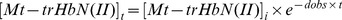
(13)


Values of *d*_on_ and *d*_off_ for isoniazid binding to Mt-trHbN(II) were determined by plotting values of *d* ^obs^ *versus* the free drug concentration (*i.e.*, [isoniazid]), according to Equation 14 [58]:
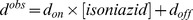
(14)


### Effect of isoniazid on O_2_ binding to Mt-trHbN(II)

Thermodynamics of O_2_ binding to Mt-trHbN(II), in the absence and presence of isoniazid, was analyzed in the framework of the minimum reaction mechanism depicted by Scheme F:
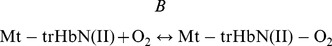
(F)


The value of the dissociation equilibrium constant (*i.e.*, *B*) for O_2_ binding to Mt-trHbN(II) was obtained spectrophotometrically between 375 nm and 460 nm, at pH 7.0 (1.0×10^−1^ M phosphate buffer) and 20.0°C.

The value of *B* was determined by the tonometer method [61] adding small volumes of air to the Mt-trHbN(II) solution (1.3×10^−6^ M), in the absence and presence of isoniazid ( =  1.0×10^−2^ M; *B* ^obs^). The O_2_ solubility in the aqueous buffered solution is 1.38×10^−3^ M, at 760.0 mmHg and 20.0°C [58]. The O_2_-dependent absorbance changes of Mt-trHbN(II) were recorded after incubation of 40 min after each addition. Test measurements performed after 2 h of Mt-trHbN(II)-O_2_ incubation ruled out slow kinetic effects. O_2_ binding to Mt-trHbN(II) was analyzed by plotting values of the molar fraction of the Mt-trHbN(II)-O_2_ drug complex (*i.e.*, α) *versus* the free O_2_ concentration (*i.e.*, [O_2_]) according to Equation 15 [58]:
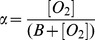
(15)
*B* changes to *B* ^obs^ in the presence of isoniazid.

Thermodynamics of competitive inhibition of O_2_ binding to Mt-trHbN(II) by isoniazid was analyzed in the framework of the minimum reaction mechanism depicted by Scheme G [59]:
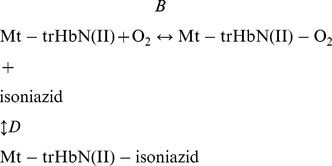
(G)


The value of the dissociation equilibrium constant for O_2_ binding to Mt-trHbN(II) in the presence of isoniazid (*B* ^obs^) was obtained at [isoniazid]  = 1.0×10^−2^ M, pH 7.0 (1.0×10^−1^ M phosphate buffer) and 20.0°C.

The inhibitory effect of isoniazid on the O_2_ affinity for Mt-trHbN(II) was analyzed according to Equation 16 [59]:
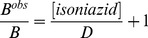
(16)


### Effect of isoniazid on CO binding to Mt-trHbN(II)

Kinetics of CO binding to Mt-trHbN(II), in the absence and presence of isoniazid, was analyzed in the framework of the minimum reaction mechanism depicted by Scheme H:
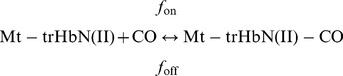
(H)


Values of the apparent pseudo-first order rate constants for CO binding to Mt-trHbN(II) (*i.e.*, *f* ^obs^) in the absence of isoniazid were determined spectrophotometrically between 375 nm and 460 nm, at pH 7.0 (1.0×10^−1^ M phosphate buffer) and 20.0°C, by rapid-mixing the CO and Mt-trHbN(II) stock solutions (3.0×10^−4^ M and 3.0×10^−6^ M, respectively) and plotting the data according to Equation 17 [58]:
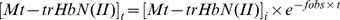
(17)


Values of the apparent pseudo-first order rate constants for CO binding to Mt-trHbN(II) (*i.e.*, *f* ^obs^) in the presence of isoniazid (*i.e.*, 5.0×10^−3^ M ≤ [isoniazid] ≥5.0×10^−2^ M) were determined spectrophotometrically between 375 nm and 460 nm, at pH 7.0 (1.0×10^−1^ M phosphate buffer) and 20.0°C, by rapid-mixing the CO and Mt-trHbN(II)-isoniazid stock solutions (3.0×10^−4^ M and 3.0×10^−6^ M, respectively). Since under some conditions the isoniazid concentration was not in large excess with respect to *D* ( =  (1.2±0.2)×10^−3^ M; see Results), the biphasic time course was analyzed according to Equation 18 [58]:
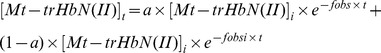
(18)where *a* and (1 – *a*) are the amplitudes of the CO binding processes to Mt-trHbN(II) in the absence and presence of isoniazid, respectively, and *f* ^obsi^ is the apparent pseudo-first order rate constant for CO binding to Mt-trHbN(II) in the presence of isoniazid.The value of *f*_on_ for CO binding to Mt-trHbN(II) in the absence and presence of isoniazid was determined by plotting values of *f* ^obs^ (or *f* ^obsi^) *versus* the free ligand concentration (*i.e.*, [CO]) according to Equation 19 [58]:



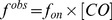
(19)The value of the first order rate constant for CO dissociation from Mt-trHbN(II)-CO (*i.e.*, *f*_off_) in the absence and presence of isoniazid ( =  5.0×10^−3^ M) was determined by CO replacement with NO, at pH 7.0 (1.0×10^−1^ M phosphate buffer) and 20.0°C. Briefly, the Mt-trHbN(II)-CO (final concentration 3.0×10^−6^ M) dithionite (final concentration, 1.0×10^−2^ M) solution was mixed with the nitrite (final concentration, 3.0×10^−3^ M) solution [69]. Kinetics was monitored at 415 nm, 420 nm, and 425 nm.

The Mt-trHbN(II)-CO decarbonylation process (*i.e.*, Mt-trHbN(II)-NO formation) was analyzed in the framework of the minimum reaction mechanism depicted by Scheme I [69]:
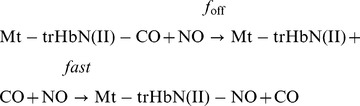
(I)


Values of *f*_off_ have been determined from data analysis according to Equation 20 [69]:
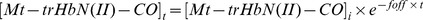
(20)


### Docking analysis

Flexible-ligand/flexible-receptor molecular docking simulation was performed by using Autodock v4.2.2.1 [70]. The structure of Mt-trHbN was downloaded from the Protein Data Bank (PDB ID code: 1IDR) [71]. The isoniazid molecule was built with Molden v5.0 [72] and its molecular geometry was optimized in gas-phase at the HF/6–31G* level of theory using GAMESS-US [73], [74]. All the input files for the molecular docking were prepared with AutoDockTools 1.5.6rc3. The docking simulation was performed on a 90 Å×90 Å×90 Å cubic grid of step 0.225 Å (20.2 Å edge) centered on the heme, using the Lamarckian genetic algorithm implemeted in AutoDock. The 250 isoniazid/flexible ligand poses so obtained were then subjected to the RMSD-based clusterization using a cut-off of 2.0 Å in order to identify representative binding conformations. The images of conformations were made with UCSF chimera 1.6.1 [75], [76].

The structure of isoniazid bound to cytosolic soybean ascorbate peroxidase was downloaded from the Protein Data Bank (PDB ID code: 2VCF) [77]. Single bonds were allowed to rotate freely during the Monte Carlo simulated annealing procedure. The analysis of the conformational space was restricted to a cubic box of 60 Å edge centered on the coordinates of heme and along the apolar tunnel systems [71]. Monte Carlo simulated annealing was performed by starting from a temperature of 900 K with a relative cooling factor of 0.95 per cycle to reach the temperature of 5 K in 100 cycles [70].

## Results

### Isoniazid binding to Mt-trHbN(III)

**Figure 1:**
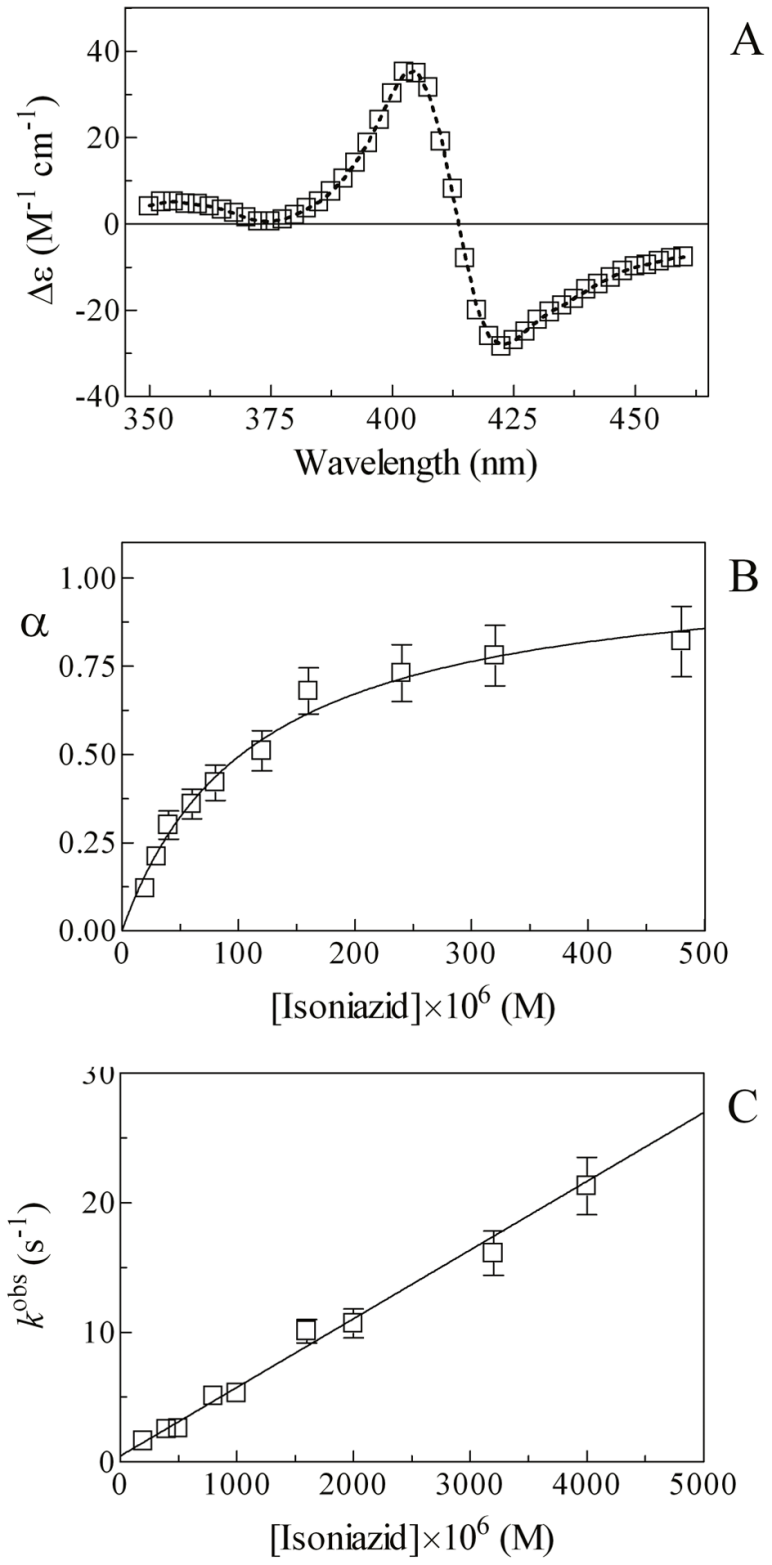
Isoniazid binding to Mt-trHbN(III). (A) Difference static and kinetic absorbance spectrum of Mt-trHbN(III) *minus* Mt-trHbN(III)-isoniazid (dotted line and squares, respectively). (B) Ligand-binding isotherm for isoniazid binding to Mt-trHbN(III). The analysis of data according to Equation 1 allowed the determination of *K* = (1.1±0.1)×10^−4^ M. (C) Dependence of the pseudo-first-order rate-constant *k* ^obs^ for isoniazid binding to Mt-trHbN(III) on the drug concentration. The analysis of data according to Equation 3 allowed the determination of *k*_on_ = (5.3±0.6)×10^3^ M^−1^ s^−1^ and *k*_off_  = (4.6±0.5)×10^−1^ s^−1^. The protein concentration was 4.0×10^−6^ M (panels A and B) and 2.0×10^−6^ M (panel C). The isoniazid concentration was 4.0×10^−3^ M (panel A). Where not shown, the standard deviation is smaller than the symbol. All data were obtained at pH 7.0 and 20.0°C. For details, see text.

Mixing of the Mt-trHbN(III) and isoniazid solutions brings about a shift of the optical absorption maximum of the Soret band (*i.e.*, λ_max_) from 406 nm (*i.e.*, Mt-trHbN(III)) to 410 nm (*i.e.*, Mt-trHbN(III)-isoniazid) and a change of the extinction coefficient from ε_406 nm_  = 1.41×10^5^ M^−1^ cm^−1^ (*i.e.*, Mt-trHbN(III)) to ε_410 nm_  = 1.09×10^5^ M^−1^ cm^−1^ (*i.e.*, Mt-trHbN(III)-isoniazid) (see Fig. 1, panel A, and Table 1). As expected for simple systems [58], the difference static and kinetic absorbance spectrum of Mt-trHbN(III) *minus* Mt-trHbN(III)-isoniazid match very well each other (Fig. 1, panel A).

**Table 1:** Values of λ_max_ and ε of the absorption spectra in the Soret region of ferric and ferrous derivatives of Mt-trHbN, at pH 7.0 and 20.0°C.

Derivative	λ_max_ (nm)	ε (M^−1^ s^−1^)
Mt-trHbN(III)	406	1.41×10^5^
Mt-trHbN(III)-isoniazid	410	1.09×10^5^
Mt-trHbN(III)-azide	415	1.28×10^5^
Mt-trHbN(II)	432	1.03×10^5^
Mt-trHbN(II)-isoniazid	420	1.33×10^5^
Mt-trHbN(II)-O_2_	416	1.07×10^5^
Mt-trHbN(II)-CO	420	1.43×10^5^

Values of λ_max_ and ε of Mt-trHbN(III), Mt-trHbN(II), Mt-trHbN(II)-O_2_, and Mt-trHbN(II)-CO are in excellent agreement with those reported in the literature [56], [57].

Over the whole isoniazid concentration range explored (from 2.0×10^−5^ M to 4.8×10^−4^ M), values of the molar fraction of the Mt-trHbN(III)-isoniazid complex are wavelength-independent, between 350 nm and 460 nm, at fixed drug concentration, however they depend on the isoniazid concentration. Isoniazid binding to Mt-trHbN(III) follows a simple equilibrium (see Scheme 1; Fig. 1, panel B). The analysis of data according to Equation 1 [58] allowed to determine the value of the dissociation equilibrium constant for isoniazid binding to Mt-trHbN(III) (*i.e.*, *K* = (1.1±0.1)×10^−4^ M). As expected for simple systems [58], the value of the Hill coefficient *n* for isoniazid binding to Mt-trHbN(III) is 1.01±0.02.

Over the whole isoniazid concentration range explored (from 2.0×10^−4^ M to 4.0×10^−3^ M), the time course for isoniazid binding to Mt-trHbN(III) corresponds to a single exponential for more than 90% of its course between 350 nm and 460 nm (Equation 2). Values of the apparent pseudo-first order rate constant for isoniazid binding to Mt-trHbN(III) (*i.e.*, *k* ^obs^) are wavelength-independent at fixed drug concentration, but they depend on the isoniazid concentration. The plot of *k* ^obs^ *versus* the isoniazid concentration is linear (see Scheme 1; Fig. 1, panel C). The analysis of data according to Equation 3 [58] allowed to determine values of the second-order association rate constant (*i.e.*, *k*_on_ = (5.3±0.6)×10^3^ M^−1^ s^−1^; corresponding to the slope of the plot) and of the first-order dissociation rate constant (*i.e.*, *k*_off_ = (4.6±0.5)×10^−1^ s^−1^; corresponding to the *y*-intercept).

As expected for simple systems [58], the value of *K* for isoniazid binding to Mt-trHbN(III) obtained at equilibrium (*K* = (1.1±0.1)×10^−4^ M; see Fig. 1, panel B) is in good agreement with that calculated from kinetic parameters (*i.e.*, *k*_off_/*k*_on_ = (8.7±1.0)×10^−5^ M; see Fig. 1, panel C).

### Effect of isoniazid on azide binding to Mt-trHbN(III)

**Figure 2:**
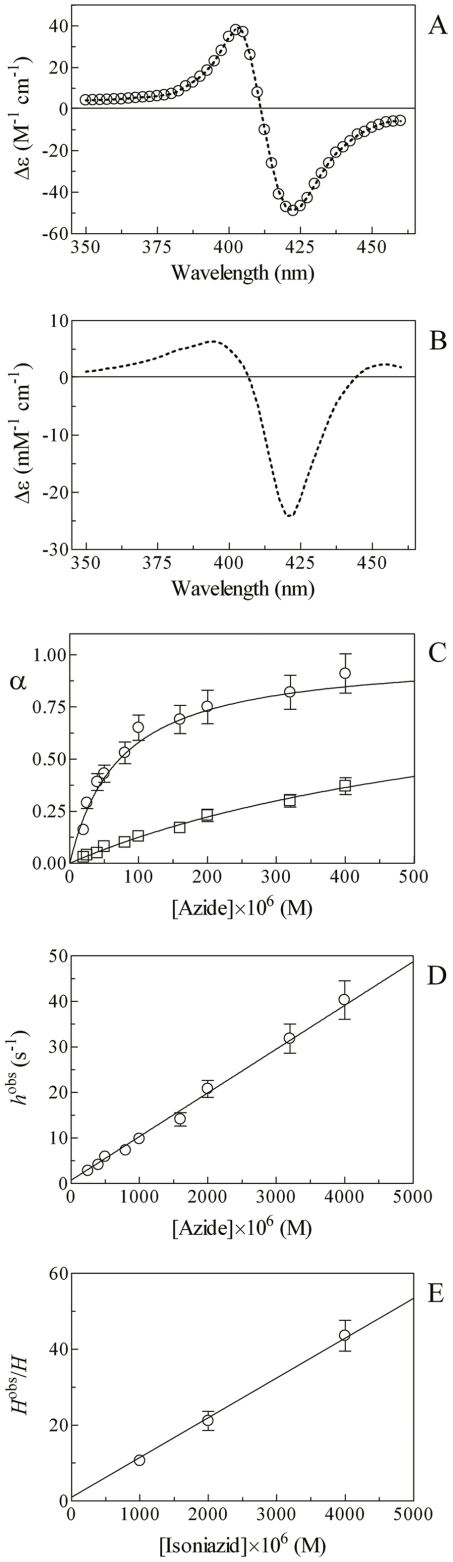
Azide binding to Mt-trHbN(III) in the absence and presence of isoniazid. (A) Difference static and kinetic absorbance spectrum of Mt-trHbN(III) *minus* Mt-trHbN(III)-azide (dotted line and open circles, respectively). (B) Difference static absorbance spectrum of Mt-trHbN(III)-isoniazid *minus* Mt-trHbN(III)-azide. (C) Ligand-binding isotherms for azide binding to Mt-trHbN(III) in the absence (circles) and presence (squares) of isoniazid. The analysis of data according to Equation 4 allowed the determination of *H* = (7.3±0.8)×10^−5^ M and *H* ^obs^  = (7.0±0.8)×10^−4^ M in the absence (circles) and presence (squares) of isoniazid. (D) Dependence of the pseudo-first-order rate-constant *h* ^obs^ for azide binding to Mt-trHbN(III) on the ligand concentration. The analysis of data according to Equation 6 allowed the determination of *h*_on_  = (9.6±1.1)×10^3^ M^−1^ s^−1^ and *h*_off_  = (7.1±0.8)×10^−1^ s^−1^. (E) Dependence of the *H* ^obs^/*H* ratio on the isoniazid concentration. The analysis of data according to Equation 7 allowed the determination of *K* = (9.5±0.9)×10^−5^ M. The protein concentration was 4.0×10^−6^ M (panels A, B, and C) and 2.0×10^−6^ M (panel D). The isoniazid concentration was 4.0×10^−3^ M (panels A and B) and 1.0×10^−3^ M (panel C). Where not shown, the standard deviation is smaller than the symbol. All data were obtained at pH 7.0 and 20.0°C. For details, see text.

In the absence of isoniazid, mixing of the Mt-trHbN(III) and azide solutions induces a shift of the optical absorption maximum of the Soret band (*i.e.*, λ_max_) from 406 nm (*i.e.*, Mt-trHbN(III)) to 415 nm (*i.e.*, Mt-trHbN(III)-azide) and a change of the extinction coefficient from ε_406 nm_  = 1.41×10^5^ M^−1^ cm^−1^ (*i.e.*, Mt-trHbN(III)) to ε_415 nm_  = 1.28×10^5^ M^−1^ cm^−1^ (*i.e.*, Mt-trHbN(III)-azide). On the other hand, in the presence of isoniazid, mixing of the Mt-trHbN(III)-isoniazid and azide solutions leads to a shift of the optical absorption maximum of the Soret band (*i.e.*, λ_max_) from 410 nm (*i.e.*, Mt-trHbN(III)-isoniazid) to 415 nm (*i.e.*, Mt-trHbN(III)-azide) and a change of the extinction coefficient from ε_410 nm_  = 1.09×10^5^ M^−1^ cm^−1^ (*i.e.*, Mt-trHbN(III)-isoniazid) to ε_415 nm_  = 1.28×10^5^ M^−1^ cm^−1^ (*i.e.*, Mt-trHbN(III)-azide) (see Fig. 2, panels A and B, and Table 1). As expected for simple systems [58], the difference static and kinetic absorbance spectra of Mt-trHbN(III) *minus* Mt-trHbN(III)-azide match very well each other (Fig. 2, panel A).

Over the whole azide concentration range explored (from 2.0×10^−5^ M to 4.0×10^−4^ M), values of the molar fraction of the Mt-trHbN(III)-azide complex are wavelength-independent, between 350 nm and 460 nm, at fixed ligand concentration, but they depend on the azide concentration. Azide binding to Mt-trHbN(III) follows a simple equilibrium (see Scheme 2; Fig. 2, panel C). The analysis of data according to Equation 4 [58] allowed to determine the value of the dissociation equilibrium constant for azide binding to Mt-trHbN(III) (*i.e.*, *H* = (7.3±0.8)×10^−5^ M). As expected for simple systems [58], the value of the Hill coefficient *n* for isoniazid binding to Mt-trHbN(III) is 0.99±0.01.

Over the whole azide concentration range explored (from 2.0×10^−4^ M to 4.0×10^−3^ M), the time course for azide binding to Mt-trHbN(III) corresponds to a single exponential for more than 95% of its course between 350 nm and 460 nm (Equation 5). Values of the apparent pseudo-first order rate constant for azide binding to Mt-trHbN(III) (*i.e.*, *h* ^obs^) are wavelength-independent at fixed ligand concentration, but they depend on the azide concentration. The plot of *h* ^obs^ *versus* the azide concentration is linear (see Scheme 2; Fig. 2, panel D). The analysis of data according to Equation 6 [58] allowed to determine values of the second-order association rate constant (*i.e.*, *h*_on_  = (9.6±1.1)×10^3^ M^−1^ s^−1^; corresponding to the slope of the plot) and of the first-order dissociation rate constant (*i.e.*, *h*_off_  = (7.1±0.8)×10^−1^ s^−1^; corresponding to the *y*-intercept) for azide binding to Mt-trHbN(III).

As expected for simple systems [58], the value of *H* for azide binding to Mt-trHbN(III) obtained at equilibrium ( =  (7.3±0.8)×10^−5^ M; see Fig. 2, panel C) is in good agreement with that calculated from kinetic parameters (*i.e.*, *h*_off_/*h*_on_  = (7.4±0.8)×10^−5^ M; see Fig. 2, panel D).

As shown in Figure 2 (panel E), isoniazid inhibits competitively azide binding to Mt-trHbN(III) (see Scheme 3). In fact, values of the *H* ^app^/*H* ratio increase linearly with the isoniazid concentration over the whole range explored (*i.e.*, 1.0×10^−3^ M ≤ [isoniazid] ≤4.0×10^−3^ M). The analysis of data according to Equation 7 [58] allowed the determination of *K* = (9.5±0.9)×10^−5^ M, corresponding to the absolute value of the *x* intercept of the linear plot. As expected for simple systems [58], the value of *K* for isoniazid binding to Mt-trHbN(III) obtained according to Equation 7 ( =  (9.5±0.9)×10^−5^ M; see Fig. 2, panel E) is in good agreement with that determined according to Equation 1 (*K* = (1.1±0.1)×10^−4^ M; see Fig. 1, panel C).

### Effect of isoniazid on peroxynitrite scavenging by Mt-trHbN(III)

Kinetics of peroxynitrite isomerization in the absence and presence of Mt-trHbN(III) and isoniazid was recorded at 302 nm. The time courses of peroxynitrite isomerization were fitted to a single-exponential decay for more than 95% of their course (Equation 8). According to the literature [63]–[70], this indicates that no intermediate species (*e.g.*, Mt-trHbN(III)-OONO; see Scheme 4) accumulate(s) in the course of peroxynitrite isomerization. Therefore, the formation of the transient Mt-trHbN(III)-OONO species represents the rate-limiting step in catalysis, the conversion of the Mt-trHbN(III)-OONO complex to Mt-trHbN(III) and NO_3_^–^ being faster by at least one-order of magnitude than its formation.

**Figure 3:**
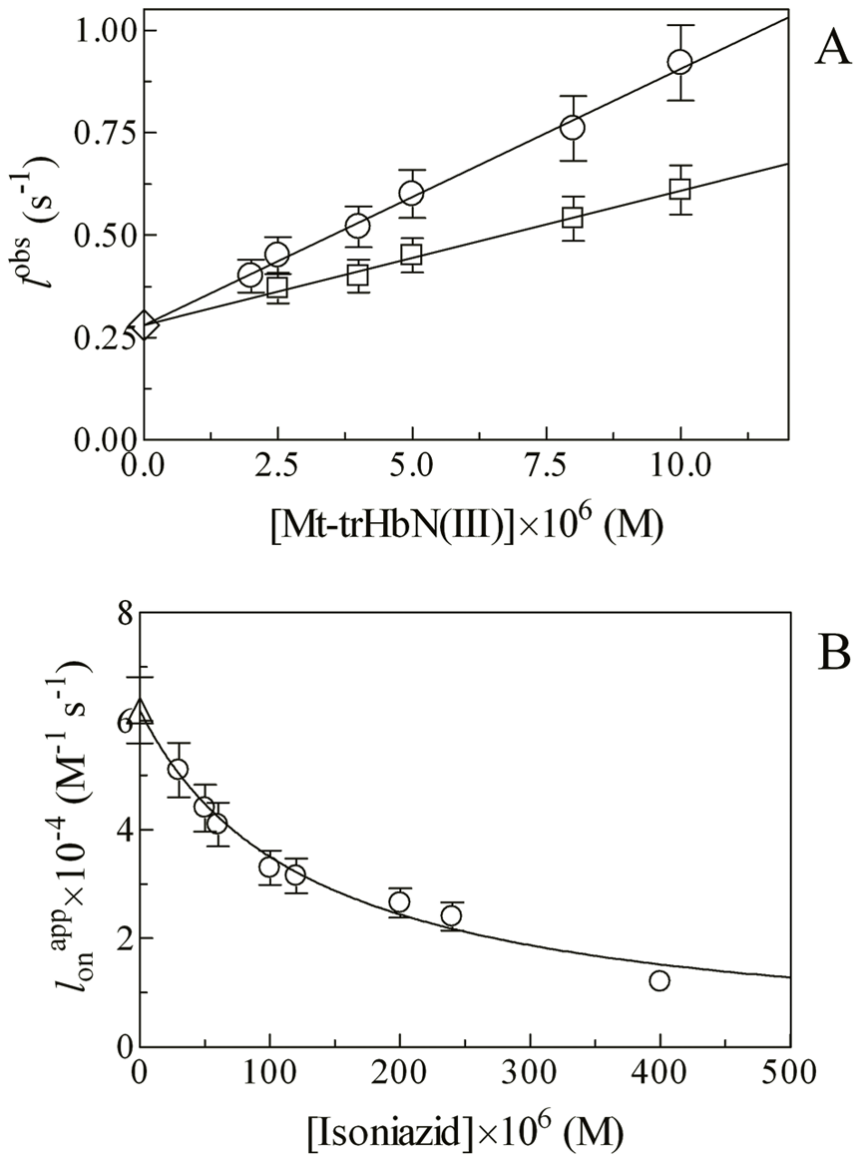
Effect of isoniazid on peroxynitrite scavenging by Mt-trHbN(III). (A) Dependence of the observed rate constant *l* ^obs^ for Mt-trHbN(III)-catalyzed isomerization of peroxynitrite on the Mt-trHbN(III) concentration, in the absence and presence of isoniazid (circles and squares, respectively). The open diamond on the ordinate indicates the average *l*_0_ value ( =  2.8×10^−1^ s^−1^). Data were analyzed according to Equation 9 with values of *l*_on_ and *l*_0_ given in Table 2. The peroxynitrite concentration was 5.0×10^−5^ M. The isoniazid concentration was 0 M (circles) and 4.0×10^−4^ M (squares). (B) Dependence of the second-order rate constant *l*_on_^app^ for Mt-trHbN(III)-catalyzed isomerization of peroxynitrite on the isoniazid concentration. The triangle on the ordinate indicates the *l*_on_ value ( =  (6.2±0.6)×10^4^ M^−1^ s^−1^) obtained in the absence of isoniazid. The analysis of data according to Equation 11 allowed the determination of *K* = (1.3±0.1)×10^−4^ M. Where not shown, the standard deviation is smaller than the symbol. All data were obtained at pH 7.0 and 20.0°C. For details, see text.

In the absence and presence of isoniazid the observed rate constant for Mt-trHbN(III)-catalyzed isomerization of peroxynitrite (*i.e.*, *l* ^obs^) increases linearly with the Mt-trHbN(III) concentration (Fig. 3, panel A). The analysis of the data reported in Figure 3 (panel A), according to Equation 9 [61] allowed the determination of values of the second-order rate constant for peroxynitrite isomerization by Mt-trHbN(III) (*i.e.*, *l*_on_, corresponding to the slope of the linear plots) and of the first-order rate constant for peroxynitrite isomerization in the absence of Mt-trHbN(III) (*i.e.*, *l*_0_; corresponding to the *y* intercept of the linear plots).

Isoniazid affects dose-dependently Mt-trHbN(III)-mediated isomerization of peroxynitrite (Fig. 3 and Table 2). Indeed, the *l*_on_ value for Mt-trHbN(III)-catalyzed isomerization of peroxynitrite decreases from 6.2×10^4^ M^−1^ s^−1^ in the absence of isoniazid to 1.2×10^4^ M^−1^ s^−1^ at the isoniazid concentration of 4.0×10^−4^ M (*i.e.*, *l*_on_^obs^). On the contrary, values of *l*_0_ are unaffected by isoniazid (see Table 2), the average *l*_0_ value being 2.8×10^−1^ s^−1^. Values of *l*_0_, obtained according to Equation 10 in the absence of Mt-trHbN(III) and in the absence and presence of isoniazid match very well each other (see Table 2) and are in excellent agreement with those reported in the literature in the absence of ferric heme-proteins [63]–[70].

**Table 2:** Effect of the isoniazid concentration on *l*_0_ and *l*_on_ values for Mt-trHbN(III)-mediated peroxynitrite isomerization, at pH 7.0 and 20.0°C.

Isoniazid (M)	*l*_on_ or *l*_on_^app^ (M^−1^ s^−1^) a	*l*_0_ (s^−1^) b
0	(6.2±0.6)×10^4^	(2.7±0.3)×10^−1^
		*(2.8±0.3)*×*10* ^−*1*^
3.0×10^−5^	(5.1±0.5)×10^4^	(2.9±0.3)×10^−1^
		*(2.7±0.3)*×*10* ^−*1*^
5.0×10^−5^	(4.4±0.4)×10^4^	(2.8±0.3)×10^−1^
		*(3.0 ±0.3)*×*10* ^−*1*^
6.0×10^−5^	(4.1±0.4)×10^4^	(3.0±0.3)×10^−1^
		*(2.6±0.3)*×*10* ^−*1*^
1.0×10^−4^	(3.3±0.3)×10^4^	(3.1±0.3)×10^−1^
		*(3.2±0.3)*×*10* ^−*1*^
1.2×10^−4^	(3.2±0.3)×10^4^	(2.6±0.3)×10^−1^
		*(2.4±0.3)*×*10* ^−*1*^
2.0×10^−4^	(2.7±0.3)×10^4^	(2.5±0.3)×10^−1^
		*(2.6±0.3)* ×*10* ^−*1*^
2.4×10^−4^	(2.4±0.3)×10^4^	(3.0±0.3)×10^−1^
		*(2.9±0.3)*×*10* ^−*1*^
4.0×10^−4^	(1.2±0.1)×10^4^	(2.5±0.3)×10^−1^
		*(2.6±0.3)*×*10* ^−*1*^

a *l*_on_ and *l*_on_^app^ indicate values of the second-order rate constant for Mt-trHbN(III)-mediated peroxynitrite isomerization obtained in the absence and presence of isoniazid, respectively.

b In regular style are shown values of *l*_0_ for Mt-trHbN(III)-catalyzed peroxynitrite isomerization. In italics are shown values of *l*_0_ for peroxynitrite isomerization obtained in the absence of Mt-trHbN(III).

According to Equation 11 [67], the analysis of the dependence of *l*_on_ for Mt-trHbN(III)-mediated isomerization of peroxynitrite on the isoniazid concentration (Fig. 3, panel B) allowed the determination of *K* for isoniazid binding to Mt-trHbN(III) ( =  (1.3±0.1)×10^−4^ M). The Hill coefficient (*n*) for isoniazid binding to Mt-trHbN(III) is 1.00±0.02, indicating that drug binding to Mt-trHbN(III) is a non-cooperative event. As expected for a simple system [61], the value of *K* determined according to Equation 11 ( =  (1.3±0.1)×10^−4^ M; Fig. 3, panel B) is in good agreement with those determined according to Equation 1 ( =  (1.1±0.1)×10^−4^ M; Fig. 1, panel A) and Equation 7 ( =  (9.5±0.9)×10^−5^ M; see Fig. 2, panel C). Under conditions where [isoniazid] >10×*K*, Mt-trHbN(III) does not catalyze the isomerization of peroxynitrite as observed in the presence of [azide] >10×*H* (*i.e.*, of the non-catalytic Mt-trHbN(III)-azide complex; see Table 3).

**Table 3:** NO_3_^–^ and NO_2_^–^ distribution of peroxynitrite isomerization in the absence and presence of Mt-trHbN(III), isoniazid, and azide, at pH 7.0 and 20.0°C.

Mt-trHbN(III) (M)	Isoniazid (M)	Azide (M)	NO_3_^–^ (%)	NO_2_^–^ (%)	NO_3_^–^ + NO_2_^–^ (%)
–	–	–	73±6	26±5	99
–	–	1.0×10^−2^	74±5	27±6	101
–	1.0×10^−2^	–	71±6	31±4	102
–	1.0×10^−2^	1.0×10^−2^	72±5	28±4	100
5.0×10^−5^	–	–	91±6	9±3	100
5.0×10^−5^	–	1.0×10^−2^	73±7	26±4	99
5.0×10^−5^	1.0×10^−2^	–	74±8	25±4	99
5.0×10^−5^	1.0×10^−2^	1.0×10^−2^	75±8	26±4	101

### Effect of isoniazid and azide on the production of the nitrogen-containing compounds by Mt-trHbN(III)-mediated peroxynitrite isomerization

According to literature [63]–[70], the spontaneous isomerization of peroxynitrite yields 73±6% NO_3_^–^ and 26±5% NO_2_^–^ in the absence of Mt-trHb(III). Moreover, isoniazid and azide do not significantly affect the NO_3_^–^ and NO_2_^–^ yields in the absence of Mt-trHbN(III) (the average yields of NO_3_^–^ and NO_2_^–^ are 72% and 29%, respectively). In the presence of Mt-trHbN(III), the NO_3_^–^ and NO_2_^–^ yields increase to 91±6% and decrease to 9±3%, respectively. On the other hand, in the presence of saturating amounts of isoniazid and azide (1.0×10^−2^ M) inhibiting Mt-trHbN(III)-mediated peroxynitrite isomerization, the average yields of NO_3_^–^ and NO_2_^–^ decrease to 74% and increase to 26%, respectively (see Table 3).

### Isoniazid binding to Mt-trHbN(II)

**Figure 4:**
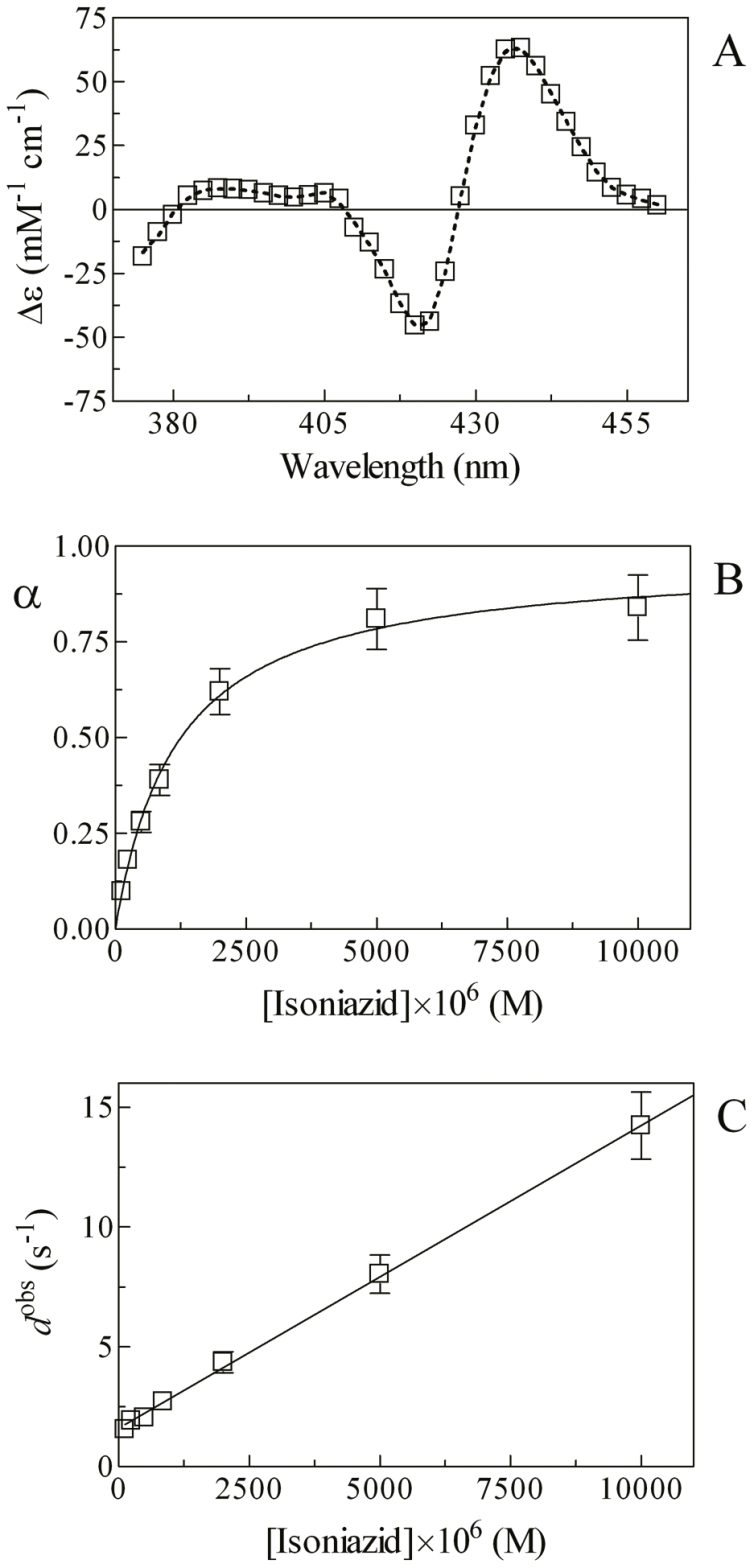
Isoniazid binding to Mt-trHbN(II). (A) Difference static and kinetic absorbance spectrum of Mt-trHbN(II) *minus* Mt-trHbN(II)-isoniazid (dotted line and squares, respectively). (B) Ligand-binding isotherm for isoniazid binding to Mt-trHbN(II). The analysis of data according to Equation 12 allowed the determination of *D* = (1.2±0.2)×10^−3^ M. (C) Dependence of the pseudo-first-order rate-constant *d* ^obs^ for isoniazid binding to Mt-trHbN(II) on the drug concentration. The analysis of data according to Equation 14 allowed the determination of *d*_on_  = (1.3±0.4)×10^3^ M^−1^ s^−1^ and *d*_off_  = 1.5±0.4 s^−1^. The protein concentration was 1.5×10^−6^ M. The isoniazid concentration was 1.0×10^−2^ M (panel A). Where not shown, the standard deviation is smaller than the symbol. All data were obtained at pH 7.0 and 20.0°C. For details, see text.

Mixing of the Mt-trHbN(II) and isoniazid solutions brings about a shift of the optical absorption maximum of the Soret band (*i.e.*, λ_max_) from 432 nm (*i.e.*, Mt-trHbN(II)) to 420 nm (*i.e.*, Mt-trHbN(II)-isoniazid) and a change of the extinction coefficient from ε_432 nm_  = 1.03×10^5^ M^−1^ cm^−1^ (*i.e.*, Mt-trHbN(II)) to ε_420 nm_  = 1.33×10^5^ M^−1^ cm^−1^ (*i.e.*, Mt-trHbN(II)-isoniazid) (see Fig. 4, panel A, and Table 1). As expected for simple systems [58], the difference static and kinetic absorbance spectra of Mt-trHbN(II) *minus* Mt-trHbN(II)-isoniazid match very well each other (Fig. 2, panel A).

Over the whole isoniazid concentration range explored (from 1.3×10^−4^ M to 2.5×10^−2^ M), values the molar fraction of the Mt-trHbN(II)-isoniazid complex are wavelength-independent, between 370 nm and 450 nm, at fixed isoniazid concentration, however they depend on the ligand concentration. Isoniazid binding to Mt-trHbN(II) follows a simple equilibrium (see Scheme 5; Fig. 4, panel B). The analysis of data according to Equation 12 [61] allowed to determine the value of the dissociation equilibrium constant for isoniazid binding to Mt-trHbN(II) (*i.e.*, *D* = (1.2±0.2)×10^−3^ M. As expected for simple systems [61], the value of the Hill coefficient *n* is 1.01±0.01.

Over the whole isoniazid concentration range explored (from 1.3×10^−4^ M to 2.5×10^−2^ M), the time course for isoniazid binding to Mt-trHbN(II) corresponds to a single exponential for more than 90% of its course between 370 nm and 450 nm (Equation 3). Values of the apparent pseudo-first order rate constant for isoniazid binding to Mt-trHbN(II) (*i.e.*, *d* ^obs^) are wavelength-independent at fixed drug concentration, but they depend on the isoniazid concentration. The plot of *d* ^obs^ *versus* the isoniazid concentration is linear (see Scheme 5; Fig. 4, panel C). Although this behavior does not necessarily mean that isoniazid is a heme ligand (keeping open the hypothesis of an isoniazid-induced conformational change, leading to hexa-coordination), the linear dependence certainly implies that: (*i*) this hexa-coordination would be much faster than isoniazid binding, and (*ii*) the overall observation is rate-limited by the interaction of isoniazid with its binding pocket. The analysis of data according to Equation 14 [61] allowed to determine values of the second-order association rate constant (*i.e.*, *d*_on_  = (1.3±0.4)×10^3^ M^−1^ s^−1^; corresponding to the slope of the plot) and of the first-order dissociation rate constant (*i.e.*, *d*_off_  = 1.5±0.4 s^−1^; corresponding to the *y*-intercept).

As expected for simple systems [61], the value of *D* for isoniazid binding to Mt-trHbN(II) obtained at equilibrium ( =  (1.2±0.2)×10^−3^ M; see Fig. 4, panel B) is in good agreement with that calculated from kinetic parameters (*i.e.*, *d*_off_/*d*_on_  = (1.2±0.2)×10^−3^ M; see Fig. 4, panel C).

### Effect of isoniazid on O_2_ binding to Mt-trHbN(II)

**Figure 5:**
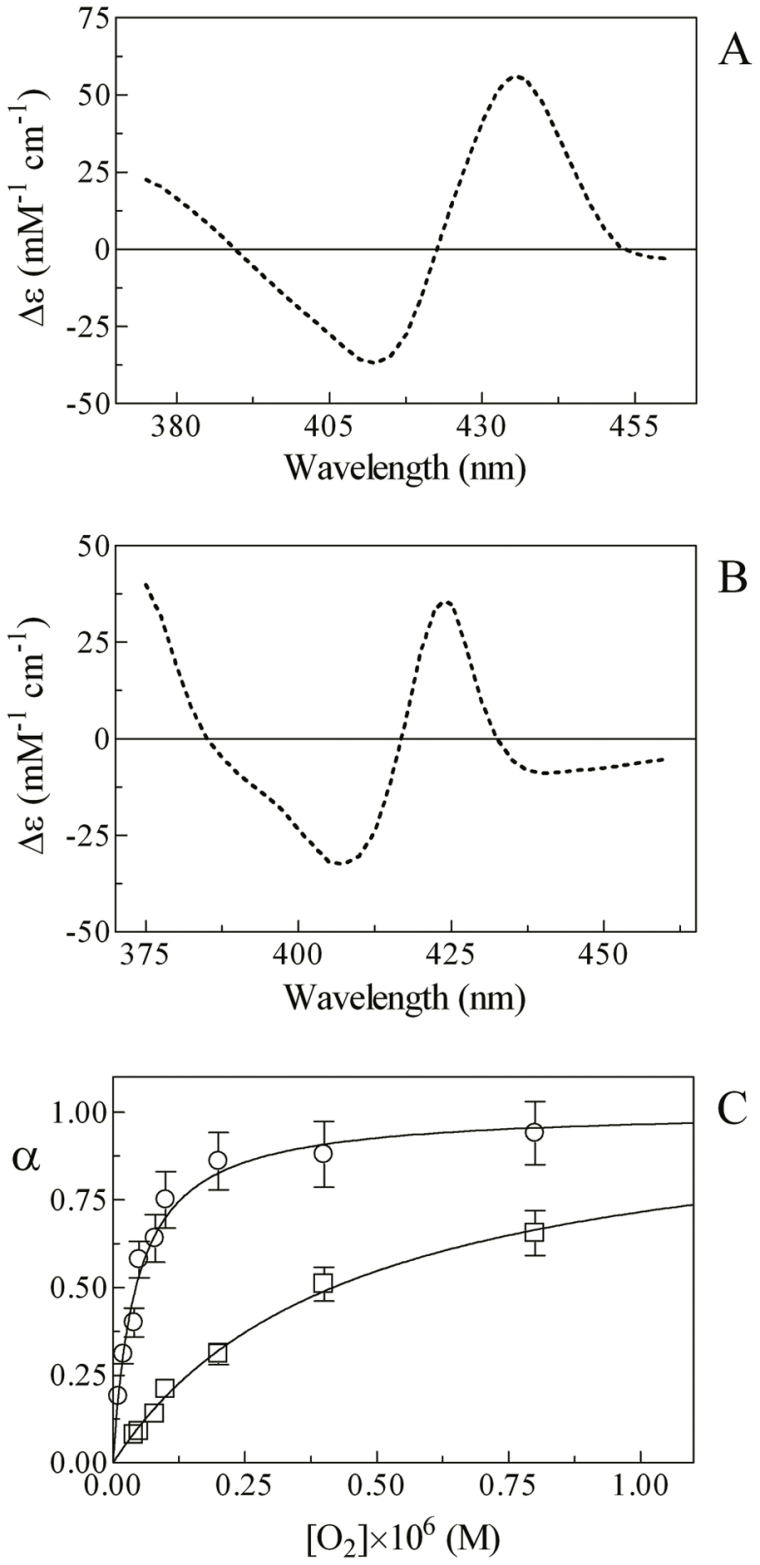
O_2_ binding to Mt-trHbN(II) in the absence and presence of isoniazid. (A) Difference static absorbance spectrum of Mt-trHbN(II) *minus* Mt-trHbN(II)-O_2_. (B) Difference static absorbance spectrum of Mt-trHbN(II)-isoniazid *minus* Mt-trHbN(II)-O_2_. (C) Ligand-binding isotherms for O_2_ binding to Mt-trHbN(II) in the absence (circles) and presence (squares) of isoniazid ( =  1.0×10^−2^ M). The analysis of data according to Equation 15 allowed the determination of *B* = (4.4±0.6)×10^−8^ M and *B* ^obs^  = (4.2±0.5)×10^−7^ M in the absence (circles) and presence (squares) of isoniazid ( =  1.0××10^−2^ M), respectively. The protein concentration was 1.3×10^−6^ M. The O_2_ concentration refers to that of the free ligand. Where not shown, the standard deviation is smaller than the symbol. All data were obtained at pH 7.0 and 20.0°C. For details, see text.

In the absence of isoniazid, adding of O_2_ to the Mt-trHbN(II) solution causes a shift of the optical absorption maximum of the Soret band (*i.e.*, λ_max_) from 432 nm (*i.e.*, Mt-trHbN(II)) to 416 nm (*i.e.*, Mt-trHbN(II)-O_2_) and a change of the extinction coefficient from ε_432 nm_  = 1.03×10^5^ M^−1^ cm^−1^ (*i.e.*, Mt-trHbN(II)) to ε_416 nm_  = 1.07×10^5^ M^−1^ cm^−1^ (*i.e.*, Mt-trHbN(II)-O_2_). On the other hand, in the presence of isoniazid, adding of O_2_ to the Mt-trHbN(II)-isoniazid solution induces a shift of the optical absorption maximum of the Soret band (*i.e.*, λ_max_) from 420 nm (*i.e.*, Mt-trHbN(II)-isoniazid) to 416 nm (*i.e.*, Mt-trHbN(II)-O_2_) and a change of the extinction coefficient from ε_420 nm_  = 1.33×10^5^ M^−1^ cm^−1^ (*i.e.*, Mt-trHbN(II)-isoniazid) to ε_416 nm_  = 1.07×10^5^ M^−1^ cm^−1^ (*i.e.*, Mt-trHbN(II)-O_2_) (see Fig. 5, panels A and B, and Table 1).

Over the whole free O_2_ concentration range explored (from 1.0×10^−8^ M to 2.0×10^−6^ M), values the molar fraction of the Mt-trHbN(II)-O_2_ complex are wavelength-independent, between 350 nm and 460 nm, at fixed O_2_ concentration; however, they depend on the O_2_ concentration. O_2_ binding to Mt-trHbN(II) follows a simple equilibrium (see Scheme 6; Fig. 5, panel C). The analysis of data according to Equation 15 [61] allowed to determine the value of the dissociation equilibrium constant for O_2_ binding to Mt-trHbN(II) (*i.e.*, *B* = (4.4±0.6)×10^−8^ M, corresponding to *P*_50_  = (2.4±0.4)×10^−2^ mm Hg). As expected for simple systems [61], the value of the Hill coefficient *n* for isoniazid binding to Mt-trHbN(II) is 1.02±0.02.

As shown in Figure 5, isoniazid inhibits competitively O_2_ binding to Mt-trHbN(II), the value of *B* ^obs^ being (4.2±0.5)×10^−7^ M, corresponding to *P*_50_  = (2.3±0.3)×10^−1^ mm Hg. According to Scheme 7 [62], the experimental value of *B* ^obs^ ( =  (4.2±0.5)×10^−7^ M; see Fig. 5, panel C) corresponds to that calculated according to Equation 16 ( =  4.1×10^−7^ M) taking into account the following parameters: [isoniazid]  = 1.0×10^−2^ M, *B* = (4.4±0.6)×10^−8^ M, and *D* = (1.2±0.2)×10^−3^ M (see Fig. 4, panel B).

### Effect of isoniazid on CO binding to Mt-trHbN(II)

**Figure 6:**
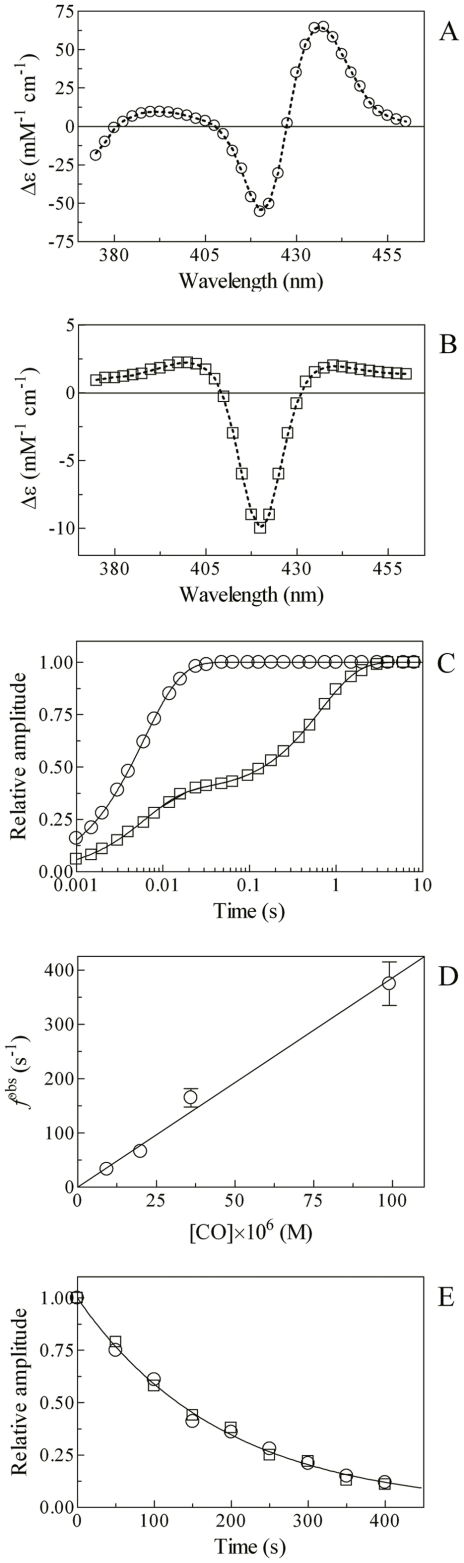
CO binding to Mt-trHbN(II) in the absence and presence of isoniazid. (A) Difference static and kinetic absorbance spectrum of Mt-trHbN(II) *minus* Mt-trHbN(II)-CO (dotted line and circles, respectively). (B) Difference static and kinetic absorbance spectrum of *Mt*-trHbN(II)-isoniazid *minus Mt*-trHbN(II)-CO (dotted line and squares, respectively). (C) Time course at λ = 421 nm of CO binding to Mt-trHbN(II) in the absence (circles) and presence (squares) of 5.0×10^−3^ M isoniazid. The analysis of data according to Equations 17 and 18, respectively, allowed the determination of the following parameters: circles – *a* = 1 and *f* ^obs^  = 1.7×10^2^ s^−1^; and squares – *a* = 0.38, *f* ^obs^  = 1.7×10^2^ s^−1^, (1 – *a*)  = 0.62, and *f* ^obsi^  = 1.5 s^−1^. (D) Dependence of the pseudo-first-order rate-constant *f* ^obs^ for CO binding to Mt-trHbN(II) on the gaseous ligand concentration. The analysis of data according to Equation 19 allowed the determination of *f*_on_  = (3.8±0.5)×10^6^ M^−1^ s^−1^. (E) Time course of CO dissociation from Mt-trHbN(II)-CO by NO replacement in the absence (circles) and presence (squares) of isoniazid ( =  5.0×10^−3^ M). The analysis of data according to Equation 20 allowed the determination of the isoniazid-independent value of *f*_off_  = (5.3±0.7)×10^−3^ s^−1^. In panels A and B, the Mt-trHbN(II), isoniazid, and/or CO concentration was 3.0×10^−6^ M, 5×10^−3^ M , and/or 3.5×10^−5^ M, respectively. Where not shown, the standard deviation is smaller than the symbol. All data were obtained at pH 7.0 and 20.0°C. For details, see text.

In the absence of isoniazid, mixing CO and Mt-trHbN(II) solutions brings about a shift of the optical absorption maximum of the Soret band (*i.e.*, λ_max_) from 432 nm (*i.e.*, Mt-trHbN(II)) to 420 nm (*i.e.*, Mt-trHbN(II)-CO) and a change of the extinction coefficient from ε_432 nm_  = 1.03×10^5^ M^−1^ cm^−1^ (*i.e.*, Mt-trHbN(II)) to ε_420 nm_  = 1.43×10^5^ M^−1^ cm^−1^ (*i.e.*, Mt-trHbN(II)-CO). On the other hand, in the presence of isoniazid, mixing CO and Mt-trHbN(II)-isoniazid solutions is not accompanied by a clear-cut shift of the optical absorption maximum of the Soret band (*i.e.*, λ_max_), since they both have a peak absorption wavelength at 420 nm; however, CO binding is accompanied by a change of the extinction coefficient from ε_420 nm_  = 1.33×10^5^ M^−1^ cm^−1^ (*i.e.*, Mt-trHbN(II)-isoniazid) to ε_420 nm_  = 1.43×10^5^ M^−1^ cm^−1^ (*i.e.*, Mt-trHbN(II)-CO) (see Fig. 6, panels A and B, and Table 1). As expected for simple systems [58], the difference static and kinetic absorbance spectra of Mt-trHbN(II) *minus* Mt-trHbN(II)-CO and Mt-trHbN(II)-isoniazid *minus* Mt-trHbN(II)-CO match very well each other (Fig. 6, panels A and B).

Over the whole CO concentration range explored (from 1.0×10^−5^ M to 1.0×10^−4^ M), the time course for CO binding to Mt-trHbN(II) in the absence of isoniazid corresponds to a single exponential for more than 90% of its course (Fig. 6, panel C) between 370 nm and 450 nm (Equation 17). Values of the apparent pseudo-first order rate constant for CO binding to Mt-trHbN(II) (*i.e.*, *f* ^obs^) in the absence of isoniazid are wavelength-independent at fixed CO concentration, but they depend on the CO concentration. The plot of *f* ^obs^ *versus* the CO concentration is linear (see Scheme 8; Fig. 6, panel D). The analysis of data according to Equation 19 [61] allowed to determine the value of the second-order association rate constant (*i.e.*, *f*_on_  = 3.8(±0.5)×10^6^ M^−1^ s^−1^; corresponding to the slope of the plot) for Mt-trHbN(II) carbonylation.

Incubation of Mt-trHbN(II) with 5.0×10^−3^ M isoniazid grossly impairs CO binding (see Fig. 6, panel C). CO binding to Mt-trHbN(II) in the presence of 5.0×10^−3^ M isoniazid is biphasic between 370 nm and 450 nm and it has been analyzed according to Equation 18. The biphasicity is due to the fact that at [isoniazid]  = 5.0×10^−3^ M Mt-trHbN(II) is only partially saturated and a significant percentage of Mt-trHbN(II) molecules have unliganded hemes, which can directly bind CO in a bimolecular fashion; thus, as expected, the fast phase corresponds to CO binding to the molar fraction of the isoniazid-free Mt-trHbN(II) (Fig 6, panel D). On the other hand, the rate of the slow phase (see Fig. 6, panel C) is independent on CO and it is characterized by a value closely similar to that of the isoniazid dissociation rate constant (*i.e.*, *d_off_*  = 1.5 s^−1^). This process corresponds to isoniazid displacement preceding CO binding and its amplitude indeed depends on the isoniazid concentration; accordingly, the final spectrum corresponds to that of Mt-trHbN(II)-CO. Notably, at high concentrations (*i.e.*, [isoniazid] ≥1.0×10^−2^ M) only the slow phase is observed (data not shown). This biphasicity indicates that the isoniazid-linked effect described on CO binding indeed must be referred to the direct binding of isoniazid to heme and not a isoniazid-induced hexa-coordination of Mt-trHbN(II). Thus, in this case the CO binding rate would have shown a isoniazid-dependent slowing down, keeping the monophasicity observed in the absence of isonazid, unless a very slow isoniazid-linked process is implied, which is ruled out by the linear dependence of the rate on isoniazid concentration (see Fig. 4, panel D).

In the absence and presence of isoniazid ( =  5.0×10^−3^ M), the time course of CO dissociation from Mt-trHbN(II)-CO by NO replacement (see Scheme 9; Fig. 6, panel E) corresponds to a single exponential for more than 90% of its course at 415 nm, 420 nm, and 425 nm (Equation 20). The analysis of data for CO dissociation from Mt-trHbN(II)-CO by NO replacement according to Equation 20 [69] allowed the determination of the isoniazid-independent value of *f*_off_  = (5.3±0.7)×10^−3^ s^−1^.

Values of *f*_on_ and *f*_off_ here determined agree with those previously reported [56].

### Automated docking simulation of isoniazid binding to Mt-trHbN

An automated docking analysis of isoniazid binding to Mt-trHbN was performed in the heme site and in the protein tunnels. The simulation shows that direct binding of isoniazid to the distal surface of the heme group turns out to be impossible due to the steric hindrance of several residues of Mt-trHbN and that the hydrophobic tunnel does not help in this respect unless a torsional degree of freedom was allowed for the side chains of residues Val28, Phe31, Tyr32, Gln57, Phe62, and Leu97. However, a possibility was found by a flexible-ligand/flexible-receptor docking simulation, where the degree of freedom of Tyr32, Phe45, and Met50 side chains has been relaxed allowing to find possible direct interactions of isoniazid with the heme.

**Figure 7:**
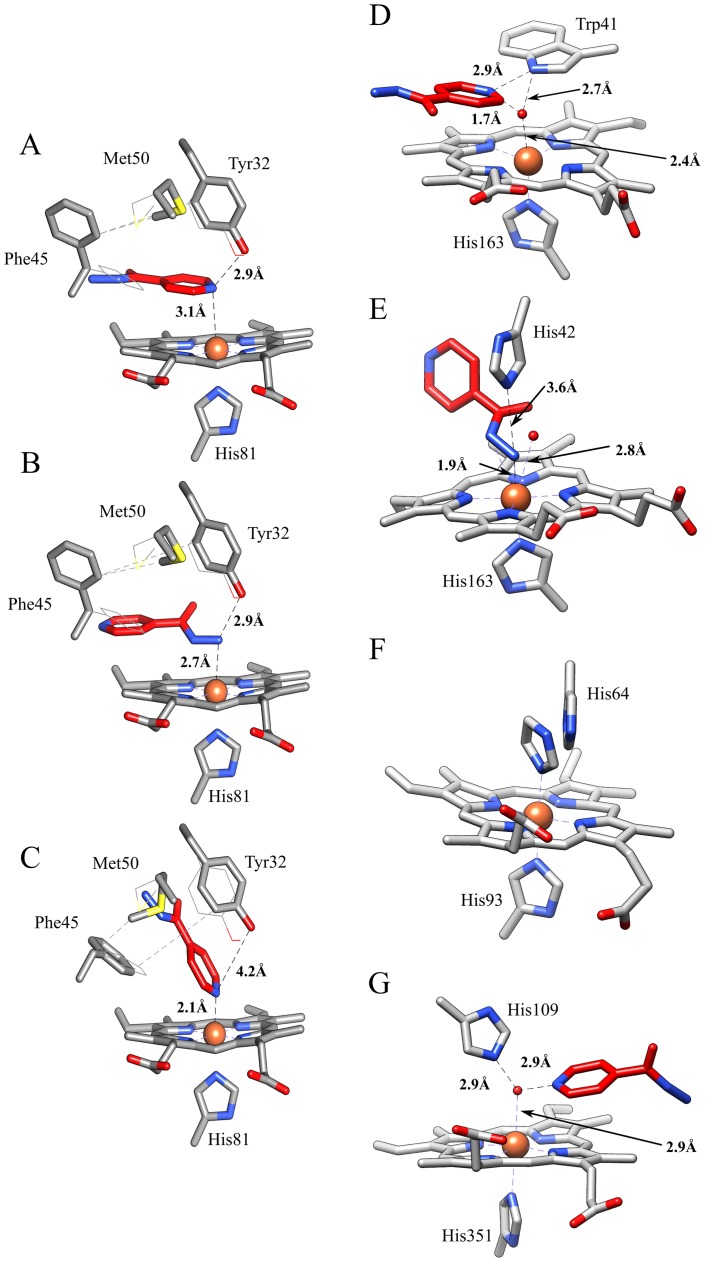
Binding modes of isoniazid to Mt-trHbN (panels A-C; present study) and related heme-protein systems (panels D-F). (A) The pyridine moiety of isoniazid is parallel to the heme plane of Mt-trHbN, and the pyridine nitrogen is at a H-bonding distance from the hydroxyl group of Tyr32. (B) The hydrazone group of isoniazid interacts with the heme-Fe atom of Mt-trHbN. (C) Isoniazid interacts with the heme-Fe atom of Mt-trHbN through the pyridine nitrogen atom. (D) Isoniazid interacts with the His42Ala mutant of sAPX by the pyridine group (PDB-ID: 2VCN). (E) Isoniazid binds to the Trp41Ala mutant of sAPX by the hydrazone group (PDB-ID: 2VCS). (F) Imidazole binding to ferric sperm whale myoglobin (PDB-ID: 1MBI). (G) Isoniazid binding to bovine lactoperoxidase (PDB-ID: 3I6N). In panels A-C, isoniazid is represented in red sticks; moreover, the heme, the proximal His81 residue, and the flexible residues Tyr32, Phe45, and Met50 are shown. Furthermore, the original conformations of the flexible residues are represented as lines. In panels D-G, only the heme, the heme proximal residue (His163, His163, His93, and His351, in panels D, E, F, and G, respectively), the heme distal residue (Trp41, His42, His64, and His109 in panels D, E, F, and G, respectively), the heme-bound ligand (isoniazid or imidazole) and the closest water molecules are shown. The distance between Tyr32 and the heme-Fe atom is not reported in panels A-C as in all the cases is longer than 5.5 Å ruling out any interaction. For details, see text.

The isoniazid-Mt-trHbN complex with the lower energy of binding is reported in Figure 7 (panel A). In this conformation, the pyridine moiety of isoniazid laid in a plane parallel to the heme, the nitrogen atom being at a coordinating distance (3.1 Å) with the heme-Fe atom, opposite to the proximal His81 residue and at H-bonding distance (2.9 Å) with the hydroxyl group of Tyr32. This complex is made possible by a torsion of the Phe45 and Met50 side chains and a slight displacement of Tyr32. The calculated binding energy of this complex is – 19.7 kJ mol^−1^ (corresponding to *K* = 4×10^−4^ M). It is very important to underline that the estimation of the binding energy for this complex, as well as for the other ones (see below), takes into account also the energy required for the structural changes of amino acid residues. As shown in Figure 7 (panel B), isoniazid may interact with the heme-Fe atom also through its hydrazone group (N3-Fe distance: 2.7 Å). Also in this case a rearrangement of Tyr32, Phe45, and Met50 residues is demanded, the calculated binding energy being −17.2 kJ mol^−1^ (corresponding to *K* = 1.1×10^−3^ M). In another pose obtained by docking, it is found that isoniazid may bind to the heme-Fe atom also with the pyridine nitrogen atom (the distance being 2.1 Å; Fig. 7, panel C) upon the rearrangement of Tyr32, Phe45, and Met50 residues. It is worth remarking that in this conformation the pyridine plane is rotated by ∼90° with respect to the imidazole plane of the proximal His81 residue. The calculated binding energy of this conformation is −13.4 kJ mol^−1^ (corresponding to *K* = 4.9×10^−3^ M), but it should be taken into account the fact that the coordination of the heme-Fe atom with the pyridine moiety of isoniazid could lead to the creation of a chemical bond, which is not considered in a classical molecular docking simulation, likely resulting in an under-estimation of the “real” binding energy of this complex.

Remarkably, the Mt-trHbN-isoniazid complexes obtained by molecular docking (Fig. 7, panels A and B) are reminiscent of those obtained by X-ray crystallography for the isoniazid-bound Trp41Ala and His42Ala mutants of soybean ascorbate peroxydase (sAPX). Indeed, in the Trp41Ala and His42Ala mutants of sAPX, isoniazid contacts the heme-Fe atom through the hydrazone group and the pyridine group, respectively (Fig. 7, panels D and E) [77]. In the sAPX His42Ala mutant (Fig. 7, panel D), isoniazid is found at H-bonding distance (2.9 Å) from the Nε atom of Trp41 and the interaction with the Fe atom appears to be mediated by a crystallographic water molecule. In the sAPX Trp41Ala mutant (Fig. 7, panel E), isoniazid fills the position occupied by the Trp41 lateral chain in the His42Ala mutant (see Fig. 7, panel D), and interacts with the heme-Fe atom through the hydrazone group (N3-Fe distance: 1.9 Å). It is worth to notice that the catalytic activity of the Trp41Ala mutant is inhibited by isoniazid [77]. Furthermore, the isoniazid binding mode to the heme-Fe atom of Mt-trHbN through the pyridine moiety (Fig. 7, panel C) is reminiscent of that reported in the case of the exogenous imidazole to penta-coordinated globins (Fig. 7, panel F) [78], and of the heme distal histidine in hexa-coordinated globins [79]–[82].

In the crystallographic structure of isoniazid-bound bovine lactoperoxidase (Fig. 7, panel G), an enzyme able to activate the prodrug, the pyridine ring is non-covalently bound on the distal heme side in a position connected with the external surface through a hydrophobic tunnel [83]. This situation is reminiscent of what observed in our flexible docking simulation, where isoniazid has been found deeply buried in the hydrophobic tunnel of Mt-trHbN; this suggests that the protein matrix tunnel may represent a possible route for the drug approach to the heme pocket.

**Figure 8:**
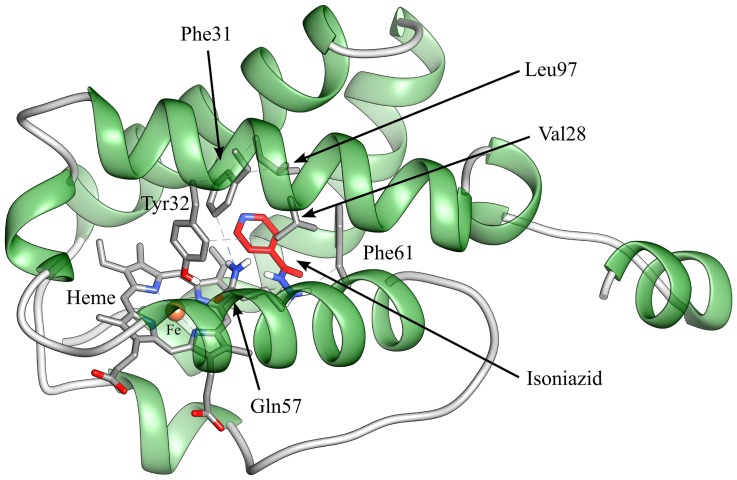
Isoniazid binding to the hydrophobic tunnel of Mt-trHbN. Mt-trHbN is represented as a green ribbon and isoniazid is depicted in red sticks. Heme and flexible residues lining the hydrophobic tunnel are shown and labelled. For details, see text.

A structure representative of non-covalent Mt-trHbN-isoniazid complexes within the tunnel is shown in Figure 8; however, it must be underlined that the tunnel should be only a passageway for isoniazid and not a binding site, since the calculated free energy of this complex is −11 kJ mol^−1^, much lower than those calculated for isoniazid binding to the heme-Fe atom.

## Discussion

The capability of mycobacteria to overcome the immune defense of the host resides mostly on the efficacy of the (pseudo)-enzymatic detoxification systems against reactive nitrogen and oxygen species. In this respect, truncated Hbs represent an important mechanism facilitating the resistance of mycobacteria to the immune response of the host [50]–[55]. Therefore, therapeutic approaches against mycobacteria may have this defense system as a target. Indeed, effective antimicrobial strategies have been developed in the past along this line, even though in recent years the emergence of antibiotic resistant strains of *M. tuberculosis* required a heavy effort to find new drugs to fight the high incidence of mycobacterial diseases against immunodepressed patients [1], [5]–[7], [9], [10].

A crucially important anti-tuberculotic drug is isoniazid, whose primary metabolic route in humans is acetylation to acetyl-isoniazid by N-acetyl-transferase. There are individual differences in the rate of isoniazid acetylation; indeed, the isoniazid acetylator phenotype of the great majority of individuals can be characterized as either slow or rapid. Individuals who are genetically rapid acetylators will have a higher acetyl isoniazid/isoniazid ratio than slower acetylators [18]. The slow or rapid acetylation of isoniazid is rarely important clinically, and a dosage reduction is only recommended for slow acetylators with hepatic failure [18]. Lastly, isoniazid inhibits the P450 system irreversibly because of binding to metabolite-intermediates [18], [84]–[86]. Therefore, the interaction of isoniazid with the defense system of *M. tuberculosis* can be of the utmost importance to develop new possible pharmacological strategies.

In this paper, we clearly demonstrate that isoniazid is able to interact with Mt-trHbN and examine the possible binding modes by docking simulations (see Fig. 7). Thus, although it cannot be excluded a priori that isoniazid might act as an allosteric effector, we report a strong evidence that isoniazid is a heme ligand for both Mt-trHbN(III) and Mt-trHbN(II), though displaying a 10-fold lower affinity for the ferrous form.

It is very interesting to remark that the Mt-trHbN-isoniazid complexes are closely similar to the sAPX-isoniazid adducts determined by X-ray crystallography and to the heme-Fe geometry of hexa-coordinated globins (see Fig. 7) [77], [79]–[82], indeed suggesting that this is a realistic representation of the isoniazid binding modes to Mt-trHbN. This seems further supported by the evidence that values of *K* and *D* calculated from the binding energy for isoniazid binding to Mt-trHbN (ranging between 4×10^−4^ M and 5×10^−3^ M) match very well with those experimentally determined (*i.e.*, *K* = 1.1(±0.1)×10^−4^ M and *D* = 1.2(±0.2)×10^−3^ M). Therefore, although it cannot excluded a priori that isoniazid might affect Mt-trHbN spectroscopic and functional properties by allosteric mechanism(s), we suggest that isoniazid is a heme ligand for both Mt-trHbN(III) and Mt-trHbN(II), though displaying a 10-fold lower affinity for the ferrous form. Furthermore, isoniazid binding inhibits Mt-trHbN(III) and Mt-trHbN(II) reactivity towards azide, CO, and O_2_, respectively. As shown in Table 4, the Mt-trHbN(III) and Mt-trHbN(II) reactivity towards azide, peroxynitrite, CO, and O_2_, is slightly higher than that of sperm whale myoglobin possibly reflecting the different geometry of the heme distal site [71]. Particularly important turns out the inhibitory effect of isoniazid on peroxynitrite detoxification, envisaging the possibility that this drug is able to effectively impair the detoxification system of *M. tuberculosis*. Lastly, isoniazid appears to bind to the interior of the protein matrix tunnel system (Fig. 8) offering a potential path for ligand diffusion to the heme distal site. This is in agreement with the diffusion and accumulation in multiple copies of ligands within the protein matrix of trHbs belonging to group N [87].

**Table 4:** Values of thermodynamic and kinetic parameters for ligand binding and peroxynitrite detoxification by *Mt*-trHbN and sperm whale Mb.

Hemeprotein	Isoniazid binding	Azide binding	Peroxynitrite detoxification
Mt-trHbN(III) a	*K* = (1.1±0.1)×10^−4^ M	*H* = (7.3±0.8)×10^−5^ M	*l*_on_ = (6.2±0.6)×10^4^ M^−1^ s^−1^
	*k*_on_ = (5.3±0.6)×10^3^ M^−1^ s^−1^	*h*_on_ = (9.6±1.1)×10^3^ M^−1^ s^−1^	
	*k*_off_ = (4.6±0.5)×10^−1^ s^−1^	*h*_off_ = (7.1±0.8)×10^−1^ s^−1^	
Mt-trHbN(III)-isoniazid a	n.a.	*H* ^obs^ = (7.0±0.8)×10^−4^ M b	*l*_on_^obs^ = (1.2±0.1)×10^4^ M^−1^ s^−1^ c
Sperm whale Mb(III)	n.d.	*H* = 5.0×10^−5^ M d	*l*_on_ = 1.6×10^4^ M^−1^ s^−1^ e
		*h*_on_ = 4.4×10^3^ M^−1^ s^−1^ d	
		*h*_off_ = 3.0×10^−1^ s^−1^ d	
KatG(III) f	*K* ∼ 1×10^−6^ M	n.d.	n.d.
	*k*_on_ = 4.8×10^2^ M^−1^ s^−1^	n.d.	n.d.
	*k*_off_ = 2.0×10^−2^ s^−1^	n.d.	n.d.
	Isoniazid binding	O_2_ binding	CO binding
Mt-trHbN(II) a	*D* = (1.2±0.2)×10^−3^ M	*B* = (4.4±0.6)×10^−8^ M	*f*_on_ = (3.8±0.5)×10^6^ M^−1^ s^−1^
	*d*_on_ = (1.3±0.4)×10^3^ M^−1^ s^−1^		*f*_off_ = (5.3±0.7)×10^−3^ s^−1^
	*d*_off_ = 1.5±0.4 s^−1^		
Mt-trHbN(II)-isoniazid a	n.a.	*B* ^obs^ = (4.2±0.5)×10^−7^ M	*f*_off_ = (5.3±0.7)×10^−3^ s^−1^
Sperm whale Mb(II)	n.d.	*B* = 5.2×10^−7^ M g	*f*_on_ = 5.1×10^5^ M^−1^ s^−1^ h
			*f*_off_ = 1.9×10^−2^ s^−1h^

a pH 7.0 and 20.0°C (present study).

b [Isoniazid]  = 1.0×10^−3^ M.

c [Isoniazid]  = 4.0×10^−4^ M.

d pH 7.0 and 25.0°C [58].

e pH 7.5 and 20.0°C [60], [61].

f pH 7.2 and 25.0°C. Of note, the *K* value calculated from values of kinetic parameters (*K*  =  *k*_off_/*k*_on_  = 4.2×10^−5^ M) differs from that determined experimentally (*K* ∼ 1×10^−6^ M) by about forty folds [38].

g pH 7.0 and 20.0°C [58].

h pH 7.0 and 20.0°C [90].

n.a., not applicable.

n.d., not determined.

The affinity of isoniazid for KatG is only apparently higher than that for Mt-trHbN. In fact, the value of *K* ( =  *k*_off_/*k*_on_) for isoniazid binding to Mt-trHbN ( =  (1.1±0.1)×10^−4^ M) (present study) is higher than that for KatG-isoniazid complex formation obtained at equilibrium (∼ 1×10^−6^ M) [38], but it is similar to that calculated from kinetic parameters ( =  *k*_off_/*k*_on_  = 4.2×10^−5^ M) [38] (Table 4). Thus, in vivo implications could be argued from the present results. Since scavenging of reactive nitrogen and oxygen species by Mt-trHbN appears to be pivotal for *M. tuberculosis* survival [50]–[54], the inhibition of Mt-trHbN(III)-catalyzed scavenging of peroxynitrite by isoniazid could represent a new action mechanism of this drug. Isoniazid could therefore play a dual role in nitrosative inhibition of *M. tuberculosis* metabolism; on one side, it can act as a primary generator of reactive nitrogen monoxide and peroxynitrite via KatG-dependent oxidation, and on the other it can impair detoxification of reactive nitrogen species by blocking the Mt-trHbN activity. Interestingly, the isoniazid dosage is 5 to 10 mg/Kg/day [18] corresponding to the 10^−5^ to 10^−4^ M plasma concentration after 1 hour from drug administration [88]. Since plasma protein binding by isoniazid is very poor [89], the isoniazid plasma concentration achievable *in vivo* [88] overlaps with the drug concentration here used (10^−5^ M to 10^−2^ M). Therefore, accounting for the average *K* and *D* values ( =  (1.1±0.2)×10^−4^ and (1.2±0.2)×10^−3^ M, respectively) here determined and the plasma level of isoniazid (10^−5^ to 10^−4^ M) [88], the molar fraction of the drug-bound Mt-trHbN could range between 1% and 50%.

Data here reported highlight the role of isoniazid as an anti-tuberculosis drug. Indeed, isoniazid not only is converted to isonicotinic acid and coupled with NADH by KatG, impairing the synthesis of the mycobacterial cell wall [26], [38], [39], but it also binds to Mt-trHbN impairing ligand binding (*e.g.*, O_2_ transport and metabolism), and peroxynitrite detoxification (present study). This last aspect appears of particular relevance since isoniazid activation by KatG produces reactive nitrogen and oxygen species that display anti-mycobacterial activity [26], [45] and are removed by mycobacterial globins including Mt-trHbN [50]–[55]. Therefore, the inhibition of the Mt-trHbN activity by isoniazid could weaken the *M. tuberculosis* survival representing a new function of this drug in the anti-tuberculosis therapy.
